# Comparative speed of kill of sarolaner (Simparica^™^) and fluralaner (Bravecto^®^) against induced infestations of *Ctenocephalides felis* on dogs

**DOI:** 10.1186/s13071-016-1373-0

**Published:** 2016-02-19

**Authors:** Robert H. Six, Julian Liebenberg, Nicole A. Honsberger, Sean P. Mahabir

**Affiliations:** Zoetis, Veterinary Medicine Research and Development, 333 Portage St., Kalamazoo, MI 49007 USA; ClinVet International (pty) Ltd, Uitsigweg, Bainsvlei 9338, Bloemfontein, Republic of South Africa

**Keywords:** Flea, *Ctenocephalides felis*, Dog, Sarolaner, Simparica^™^, Fluralaner, Bravecto^®^, Oral, Speed of kill

## Abstract

**Background:**

Fleas are the most common ectoparasite infesting dogs globally and cause direct discomfort, induce allergic reactions, and transmit pathogenic agents. Rapid speed of kill is an important characteristic for a parasiticide in order to alleviate the direct deleterious effects of fleas, reduce the impact of allergic responses, and break the flea life cycle. In this study, the speed of kill of a novel, orally administered isoxazoline parasiticide, sarolaner (Simparica^™^), against fleas on dogs was evaluated and compared with fluralaner (Bravecto^®^) over a 3-month period.

**Methods:**

Based on pretreatment flea counts, 24 dogs were randomly allocated to treatment with oral sarolaner at the label rate (2 to 4 mg/kg), once a month for 3 months, or oral fluralaner (25 to 50 mg/kg), once per label directions, or placebo. Dogs were combed and live fleas counted at 8, 12, and 24 h after treatment and subsequent re-infestations on Days 14, 29, 44, 59, 74 and 90. Efficacy was determined at each time point relative to counts for placebo dogs.

**Results:**

There were no adverse reactions to treatment. Three monthly doses of sarolaner provided ≥97.6 % efficacy (based on arithmetic means) within 8 h of treatment or subsequent weekly re-infestations of fleas for 3 months. By 12 h, fleas were eradicated from all dogs (100 % efficacy). Significantly greater numbers of live fleas were recovered from fluralaner-treated dogs at 8 h on Days 74 and 90 (*P* ≤ 0.0043) when efficacy (based on arithmetic means) was only 80.7 and 72.6 %, respectively.

**Conclusions:**

In this controlled laboratory evaluation**,** sarolaner had a significantly faster speed of kill against fleas than fluralaner at the end of its claimed treatment period. The rapid and consistent kill of fleas within 8 to 12 h after monthly oral doses of sarolaner indicates that this treatment will provide rapid and highly effective control of flea infestations, and suggests that it will provide relief for dogs suffering from flea allergy dermatitis, and should reduce the risk of flea-borne pathogen transmission.

## Background

The cat flea, *Ctenocephalides felis felis,* is the most common ectoparasite of dogs and cats worldwide [1, 2]. In addition to direct irritation and other deleterious effects of their blood-feeding, they cause flea allergic dermatitis (FAD), which is the major dermatologic disease of dogs [3]. Fleas also serve as the vector of various bacterial pathogens and are the intermediate host for filarioid and cestode parasites [4–9].

Flea infestations of pets and the home are a common occurrence and their control and elimination is often a challenge [1]. Fleas feed almost immediately on attaining a host [10], and direct irritation and allergic reactions are dependent upon the frequency and duration of feeding [11]. Also, the longer that a host is exposed to feeding fleas, the greater the chance of the transmission of flea-borne pathogens. Therefore, the rapid kill and removal of live fleas from the host is essential to ameliorate the direct effects of flea infestations and the associated adverse clinical conditions and diseases they can cause. Highly effective topical and systemic parasiticides have allowed the primary means of flea control to be through the direct treatment of the pet. These have largely eliminated the need to treat the environment, and their wide spread use as host-targeted therapies has reduced the severity and prevalence of FAD [2]. Recently, a new class of compounds, the isoxazolines, have shown excellent efficacy against both fleas and ticks for a month or longer following a single orally administered dose [12, 13].

Sarolaner (Simparica^™^, Zoetis) is a new isoxazoline effective against fleas and ticks for one month following a single dose. A laboratory study was conducted to compare the efficacy and speed of kill of three monthly doses of sarolaner (Simparica^™^) with a single oral dose of fluralaner (Bravecto^®^, Merck) against an existing flea (*C. felis*) infestation and subsequent re-infestations over a period of 3 months.

## Methods

### Ethical approval

The study was a masked, negative controlled, randomized, laboratory efficacy design conducted in the Republic of South Africa. Procedures were in accordance with the World Association for the Advancement of Veterinary Parasitology (WAAVP) guidelines for evaluating the efficacy of parasiticides for the treatment, prevention and control of flea and tick infestation on dogs and cats [14] and complied with the principles of Good Clinical Practices [15]. The protocol was reviewed and approved by the local Institutional Animal Care and Use Committee. Masking of the study was assured through the separation of functions. All personnel conducting observations or animal care or performing infestations and counts were masked to treatment allocation.

### Animals

Twenty-four, male and female, purpose-bred dogs (one Beagle and 23 mongrels) ranging in age from 12 to 76 months, and weighing from 9.6 to 29.2 kg were used in the study. Each dog was individually identified by electronic transponder. All dogs had undergone an adequate wash-out period to ensure that no residual ectoparasiticide efficacy remained from any previously administered compound. The dogs were acclimatized to study conditions for a minimum of 14 days before the treatment on Day 0. Dogs were individually housed in indoor runs such that no physical contact was possible between them. Dogs were fed an appropriate maintenance ration of a commercial dry canine feed, and water was available *ad libitum*. All dogs were given a physical examination to evaluate general health and suitability for inclusion into the study. General health observations were performed at least once daily from the start of the acclimation period to the end of the study.

### Design

The study followed a randomized complete block design. Dogs were ranked according to decreasing pretreatment flea counts into blocks of three and within each block a dog was randomly allocated to treatment with either placebo or sarolaner on Days 0, 30 and 60, or to treatment with fluralaner on Day 0 followed by placebo on Days 30 and 60 (to maintain masking). There were eight dogs per treatment group. Dogs were infested with fleas one day prior to the initial treatment and then at approximately 14-day intervals (Days 14, 29, 44, 59, 74 and 90) for 3 months. Flea counts were conducted at 8, 12, and 24 h after the initial treatment and each subsequent re-infestation. The 24-hour flea counts on Days 30 and 60 were conducted prior to treatment administration.

### Treatment

Day -1, 29, and 59 bodyweights were used to determine the appropriate doses to be administered on Days 0, 30, and 60, respectively. On each treatment day, dogs received either a placebo tablet, the appropriate strength Simparica^™^ chewable tablet to provide sarolaner at the recommended label dose of 2 mg/kg (range: 2 to 4 mg/kg), or Bravecto^®^ only on Day 0, per label directions (fluralaner at 25 to 50 mg/kg). On days 30 and 60, dogs in the fluralaner group were administered placebo. All doses were administered by hand pilling to ensure accurate and complete dosing. Each dog was observed for several minutes after dosing for evidence that the dose was swallowed, and for general health at 1, 4, and 24 h after treatment administration. In order to comply with Bravecto^®^ label requirements, all dogs were offered their regular ration within 30 min of treatment.

### Flea infestation and assessment

The *C. felis* used in the study were from a locally maintained laboratory colony of European origin. The colony was initiated in 2010 with fleas from Germany. Additional fleas obtained from Ireland were introduced into the colony in 2012 (approximately two years prior to the study). Flea infestations were performed on Days -7 (host suitability and allocation), -1, 14, 29, 44, 59, 74, and 90. At each infestation a pre-counted aliquot of 100 (±5) adult, unfed *C. felis* were directly applied to the animal which was gently restrained for a few minutes to allow the fleas to penetrate and disperse into the hair coat. Each dog was examined and combed to remove and count fleas at 24 h after the initial host suitability infestation, and at 8, 12, and 24 (±1) hours after treatment and each subsequent weekly re-infestation. Fleas were replaced on the dogs immediately after each 8 and 12 h evaluation, and discarded after the 24 h counts.

Flea counts were performed by personnel trained in the standard procedures in use at the test facility. Commercial fine-toothed flea combs were used. Dogs were combed using repeated strokes initially while standing, starting from the head, then proceeding caudally along the dorsum. The dog was then placed on each side and then on its back for combing of the sides and ventral surfaces. After a few combing strokes were completed, the comb was examined and hair and fleas were removed from the comb and all live fleas were counted. Dogs were repeatedly combed for a minimum of 10 min; if any fleas were recovered in the last minute, combing was continued in one-minute increments until no fleas were detected.

### Statistical analysis

The individual dog was the experimental unit and the primary endpoint was the live flea count. Data for post-treatment flea counts were summarized with arithmetic (AM) and geometric (GM) means by treatment group and timepoint. Flea counts were transformed (log_e_(count + 1)) prior to analysis to stabilize the variance and normalize the data. Using the PROC MIXED procedure (SAS 9.2, Cary NC), transformed counts were analyzed using a mixed linear model. The fixed effects were treatment, timepoint and the interaction between timepoint and treatment by timepoint. The random effects included room, block within room, block by treatment interaction, and error. Testing was two-sided at the significance level α = 0.05.

The assessment of efficacy was based on the percent reduction in the AM and GM live flea counts relative to placebo calculated using Abbott’s formula:

$$ \%\ \mathrm{reduction} = 100 \times \frac{\mathrm{mean}\ \mathrm{count}\ \left(\mathrm{placebo}\right)\hbox{--} \mathrm{mean}\ \mathrm{count}\ \left(\mathrm{treated}\right)}{\mathrm{mean}\ \mathrm{count}\ \left(\mathrm{placebo}\right)} $$

## Results

There were no treatment-related adverse events during the study. Placebo-treated dogs maintained good flea infestations throughout the study and these counts were maintained even following the combing and re-infestation procedures at 8 and 12 h (Tables 1, 2 and 3).

**Table 1:** Mean live flea counts and efficacy relative to placebo at 8 hours after initial treatment and post-treatment re-infestations for dogs treated with three monthly oral doses of sarolaner or a single oral dose of fluralaner

Treatment		Day of initial treatment or re-infestation^1^						
		0	14	29	44	59	74	90
Placebo	Range	44–94	71–86	39–96	48–93	35–91	58–93	30–71
	A. mean	75.0	75.5	73.1	70.8	72.5	72.4	56.1
	G. mean^2^	73.3^a^	75.4^a^	70.2^a^	69.0^a^	69.5^a^	71.4^a^	54.5^a^
Sarolaner	Range	0–0	0–0	0–8	0–0	0–2	0–0	0–1
	A. mean	0.0	0.0	1.8	0.0	0.5	0.0	0.1
	Efficacy (%)	100	100	97.6	100	99.3	100	99.8
	G. mean^2^	0.0^b^	0.0^b^	1.0^b^	0.0^b^	0.4^b^	0.0^c^	0.1^c^
	Efficacy (%)	100	100	98.6	100	99.5	100	99.8
	*P*-value vs. placebo	<0.0001	<0.0001	<0.0001	<0.0001	<0.0001	<0.0001	<0.0001
Fluralaner	Range	0–0	0–1	0–4	0–0	0–19	0–54	0–63
	A. mean	0.0	0.1	0.6	0.0	2.6	14.0	15.4
	Efficacy (%)	100	99.8	99.1	100	96.4	80.7	72.6
	G. mean^2^	0.0^b^	0.1^b^	0.3^b^	0.0^b^	0.7^b^	7.1^b^	4.0^b^
	Efficacy (%)	100	99.9	99.5	100	99.0	90.0	92.7
	*P*-value vs. placebo	<0.0001	<0.0001	<0.0001	<0.0001	<0.0001	<0.0001	<0.0001
	*P*-value vs. sarolaner	1.000	0.3311	0.2334	1.0000	0.4991	<0.0001	0.0043

^1^Sarolaner administered on Days 0, 30, 60; fluralaner on Day 0 only

^2^Geometric means within columns with the same superscript are not significantly different (*P* > 0.05)

**Table 2:** Mean live flea counts and efficacy relative to placebo at 12 hours after initial treatment and post-treatment re-infestations for dogs treated with three monthly oral doses of sarolaner or a single oral dose of fluralaner

Treatment		Day of treatment or re-infestation^1^						
		0	14	29	44	59	74	90
Placebo	Range	39–90	54–74	30–91	45–87	37–81	45–82	21–71
	A. mean	68.0	65.3	63.8	65.6	66.9	64.1	50.5
	G. mean^2^	66.1^a^	64.9^a^	60.2^a^	63.5^a^	65.2^a^	63.0^a^	47.4^a^
Sarolaner	Range	0–0	0–0	0–0	0–0	0–0	0–0	0–0
	A. mean	0.0	0.0	0.0	0.0	0.0	0.0	0.0
	Efficacy (%)	100	100	100	100	100	100	100
	G. mean^2^	0.0^b^	0.0^b^	0.0^b^	0.0^b^	0.0^b^	0.0^b^	0.0^b^
	Efficacy (%)	100	100	100	100	100	100	100
	*P*-value vs. placebo	<0.0001	<0.0001	<0.0001	<0.0001	<0.0001	<0.0001	<0.0001
Fluralaner	Range	0–0	0–0	0–0	0–0	0–1	0–17	0–15
	A. mean	0.0	0.0	0.0	0.0	0.1	2.5	2.3
	Efficacy (%)	100	100	100	100	99.8	96.1	95.5
	G. mean^2^	0.0^b^	0.0^b^	0.0^b^	0.0^b^	0.1^b^	0.7^b^	0.8^b^
	Efficacy (%)	100	100	100	100	99.9	98.9	98.4
	*P*-value vs. placebo	<0.0001	<0.0001	<0.0001	<0.0001	<0.0001	<0.0001	<0.0001
	*P*-value vs. sarolaner	1.000	1.000	1.000	1.0000	0.4040	0.1128	0.0630

^1^Sarolaner administered on Days 0, 30, 60; fluralaner on Day 0 only

^2^Geometric means within columns with the same superscript are not significantly different (*P >* 0.05)

**Table 3:** Mean live flea counts and efficacy relative to placebo at 24 hours after initial treatment and post-treatment re-infestations for dogs treated with three monthly oral doses of sarolaner or a single oral dose of fluralaner

Treatment		Day of treatment or re-infestation^1^						
		0	14	29	44	59	74	90
Placebo	Range	33–91	43–69	29–89	41–73	38–77	38–75	12–74
	A. mean	64.6	56.8	56.1	55.8	61.9	55.6	46.9
	G. mean^2^	62.3^a^	56.0^a^	53.4^a^	54.0^a^	60.7^a^	54.4^a^	41.5^a^
Sarolaner	Range	0–1	0–0	0–0	0–0	0–0	0–0	0–0
	A. mean	0.1	0.0	0.0	0.0	0.0	0.0	0.0
	Efficacy (%)	99.8	100	100	100	100	100	100
	G. mean^2^	0.1^b^	0.0^b^	0.0^b^	0.0^b^	0.0^b^	0.0^b^	0.0^b^
	Efficacy (%)	99.9	100	100	100	100	100	100
	*P*-value vs. placebo	<0.0001	<0.0001	<0.0001	<0.0001	<0.0001	<0.0001	<0.0001
Fluralaner	Range	0–0	0–0	0–0	0–0	0–0	0–0	0–0
	A. mean	0.0	0.0	0.0	0.0	0.0	0.0	0.0
	Efficacy (%)	100	100	100	100	100	100	100
	G. mean^2^	0.0^b^	0.0^b^	0.0^b^	0.0^b^	0.0^b^	0.0^b^	0.0^b^
	Efficacy (%)	100	100	100	100	100	100	100
	*P*-value vs. placebo	<0.0001	<0.0001	<0.0001	<0.0001	<0.0001	<0.0001	<0.0001
	*P*-value vs. sarolaner	0.4444	1.0000	1.0000	1.0000	1.0000	1.0000	1.0000

^1^Sarolaner administered on Days 0, 30, 60; fluralaner on Day 0 only

^2^Geometric means within columns with the same superscript are not significantly different (*P >* 0.05)

At the 8-hour time point, both treatments resulted in significantly lower flea counts than placebo-treated dogs (*P* < 0.0001) throughout the study (Table 1). Treatment with sarolaner also resulted in significantly lower flea counts than fluralaner at 8 h on Days 74 and 90 (*P* ≤ 0.0043). The sarolaner treatment provided consistent, high efficacy at 8 h (≥98.6 %, ≥97.6 % GM, AM) for the entire study. Efficacy for fluralaner declined to 90.0 % and 92.7 % (GM), and to 80.7 % and 72.6 % (AM) on Days 74 and 90, respectively (Table 1). Over 50 live fleas were found on a fluralaner-treated dog on Days 74 and 90, while a maximum of only eight live fleas were collected from a single sarolaner-treated dog on Day 29. Sarolaner-treated dogs were free of fleas on Day 74 and only a single live flea was recovered on Day 90 (efficacy of 99.8 %, AM and GM).

**Fig. 1:**
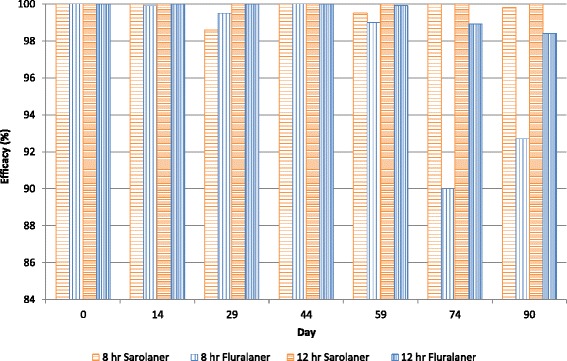
Percent efficacy based on geometric mean counts relative to placebo at 8 and 12 hours after initial treatment and post-treatment re-infestations of fleas for dogs treated with three monthly oral doses of sarolaner or a single oral dose of fluralaner

At the 12-hour timepoint, both treatments resulted in significantly lower flea counts than placebo-treated dogs (*P* < 0.0001) throughout the study (Table 2). At 12 h, flea counts were not significantly different between sarolaner- and fluralaner-treated dogs on any day (*P* ≥ 0.0630). Efficacy was consistently high for sarolaner with all dogs being free of fleas within 12 h after treatment and re-infestations through Day 90. For fluralaner, 100 % efficacy was achieved within 12 h after treatment and reinfestations up to Day 44. However, efficacy in the final month of the 3-month treatment period (Fig. 1) declined to 98.9 % and 98.4 % (GM) and 96.1 % and 95.5 % (AM) on Days 74 and 90, respectively (Table 2). As many as 17 live fleas were found on fluralaner-treated dogs at the 12-hour counts on Days 74 and 90.

At the 24-hour time point, both treatments resulted in significantly lower flea counts than placebo-treated dogs (*P* < 0.0001) throughout the study (Table 3). Both products provided ≥99.8 % reduction in mean live flea counts through Day 90 with 100 % efficacy at most evaluations (Table 3); there was no difference between the counts for the two products on any assessment day (*P* ≥ 0.4444).

## Discussion

The initial speed of kill of a parasiticide is important to provide the pet with rapid relief from the existing infestation, and rapid killing of new flea infestations consistently throughout the treatment period is critical to assist in the management of flea allergic dermatitis, to eliminate fleas before they can reproduce, and to decrease the likelihood of vector-borne pathogen transmission. Three monthly oral doses of sarolaner at the label rate of 2 to 4 mg/kg resulted in the rapid reduction of an existing flea infestation after the first dose, and the rapid and consistent kill of newly infested fleas for 90 days. Treatment with sarolaner resulted in effective flea control of greater than 97.6 % (AM) within 8 h after treatment and re-infestations for 90 days, and eliminated fleas from all dogs within 12 h over the entire 90 days. This rapid and consistent efficacy will disrupt the flea life cycle by killing adult fleas before they can lay eggs, thus reducing the environmental infestation level [16, 17].

The efficacy provided by fluralaner in the last half month of its 3-month claimed treatment period was lower and it required up to 24 h to eradicate fleas following re-infestations on Days 59, 74, and 90. Significantly more live fleas were found on fluralaner-treated dogs (up to 63 fleas/dog) than on sarolaner-treated dogs (a single flea on one dog) on Days 74 and 90 (*P* ≤ 0.0043) at the 8-hour time point. This pattern reflects the treatment intervals for the two products; sarolaner as a monthly administered tablet, and fluralaner as single dose to provide efficacy for up to 3 months. The relatively high dose required to achieve the persistent efficacy of fluralaner results in rapid speed of kill of fleas in the initial part of the treatment period, but this declines significantly relative to the efficacy of monthly sarolaner towards the end of the 3-month treatment period, while sarolaner maintains rapid and consistent speed of kill. To ensure that veterinarians and owners can be confident of protection against fleas, consistent and rapid speed of kill is essential for the entire treatment period. The decline in speed of kill for fluralaner in the last few weeks of the 3-month treatment period results in a possible gap in protection that could increase the risk of FAD or the transmission of flea-borne pathogens relative to a monthly treatment such as sarolaner, which provides consistent speed of kill for the entire treatment period.

The rapid and consistent speed of kill of fleas over a period of 30 days makes sarolaner an excellent option for flea control that will probably reduce the direct irritation caused by flea infestation, will assist in preventing the clinical signs of FAD, and should reduce the risk of flea-borne infections and diseases.

## Conclusions

Three monthly doses of sarolaner provided rapid and consistent control of existing infestations and re-infestations of fleas from 8 h onwards. The speed of kill of fluralaner however declined significantly relative to that of sarolaner during the last half month, with significantly more fleas found on fluralaner-treated dogs at 8 h after infestations on Days 74 and 90. This superior speed of kill of sarolaner at the end of the 3 months, combined with its consistent efficacy, indicates that monthly treatment with sarolaner chewable tablets (Simparica^™^) may provide more consistent protection against fleas and will probably reduce the deleterious effects of flea infestation, prevent the clinical signs of FAD, and reduce the risk of flea-borne diseases.
